# 

**Published:** 1889-08

**Authors:** 


CONTENTS.

VOL. 36 No. 8.

| | PAGE |
|---|---|
| How New Yorkers are Housed | 169 |
| Suicide | 170 |
| Health Without Medicine | 174 |
| Th Poison of Tobacco | 177 |
| Statistics of Breathing | 178 |
| Pure Instinct | 179 |
| Was it a Vision | 179 |
| How Passover Bread is Made | 180 |
| What are the Thoughts of the Dying | 180 |
| Searching for a Soul | 181 |
| Catalepsy | 182 |
| Apoplexy | 183 |
| Not that Kind of a God | 183 |
| Why Women get so Short of Breath | 184 |
| Save Your Strength | 185 |
| Lincoln's Religion | 185 |
| "Christian Science" Cures | 186 |
| A Test for Te | 186 |
| Lager Beer as Beverage | 187 |
| A Cure for Sweating Feet | 187 |
| Workingmen and Drink | 188 |
| Disease in the Sewing Machine | 188 |
| Book Notices | 189 |
| Household | 190 |
| Miscellaneous | 191 |

INDEX TO ADVERTISEMENTS OF HALF A PAGE AND OVER.

| | PAGE. |
|---|---|
| The Consumers' Supply Ass'n. | I |
| Celluloid | II |
| Hollow Suppositories | III |
| E. C. Morris & Co | IV |
| Lactopeptine | V |
| An Unprecedented Offer | VI |
| Revised Premium List | VIII |
| The Herb Cure Co | X |
| Ladies' Home Companion | XI |

